# Asynchronous Bilateral Obturator Hernias: A Suggested Approach to Reduce Morbidity of Obturator Hernias

**DOI:** 10.7759/cureus.5260

**Published:** 2019-07-28

**Authors:** David Rubay, Levonti Ohanisian, Rebecca Shin, Boris Hristov, Jose Yeguez

**Affiliations:** 1 Surgery, Charles E. Schmidt College of Medicine, Florida Atlantic University, Boca Raton, USA; 2 Orthopaedic Surgery, Charles E. Schmidt College of Medicine, Florida Atlantic University, Boca Raton, USA; 3 Surgery, Florida Atlantic University School of Medicine, Boca Raton, USA

**Keywords:** obturator hernia, asynchronous

## Abstract

Obturator hernias (OHs) are rare pelvic hernias that involve the protrusion of intraperitoneal or extraperitoneal organs or tissues through the obturator foramen. Risk factors for OH patients include female gender, chronic disease, age, malnourishment, history of multiple pregnancies, anatomical enlargement of obturator foramen, increased intraabdominal pressure, and defective collagen metabolism. Since OHs have the highest mortality rate of all abdominal hernias, prompt diagnosis and treatment are critical. Prior research has demonstrated an increased likelihood of bilateral OHs relative to unilateral. We present the case of a 79-year-old female who presented with an obstructed OH six months after an operation for an OH on the contralateral side. Due to the potential morbidity and mortality associated with OHs and delay in discovery, we suggest evaluation and treatment of the contralateral side in patients who present with unilateral OHs.

## Introduction

Obturator hernias (OHs) are rare pelvic hernias that involve the protrusion of intraperitoneal or extraperitoneal organs or tissues through the obturator foramen [1]. The incidence of OHs ranges from 0.073% to 1% of all hernias and 0.2% to 1.6% of all small bowel obstructions [2]. OHs are typically found in multiparous and emaciated elderly females with chronic diseases. It is more frequently found on the right side and 6% of cases are bilateral [3].

Approximately two-thirds of OHs are not diagnosed until exploratory laparotomy/laparoscopy. Given the nonspecific symptoms and clinical findings, early explorative surgery is suggested as an accurate and definitive diagnosis and treatment. Minimally invasive laparoscopic surgery is commonly performed, which is also useful for bilateral visualization of the obturator spaces [4]. Out of all abdominal hernias, OHs have the highest mortality rate that can be as high as 70%, depending on when it is discovered [5]. Therefore, rapid diagnosis and treatment is critical to prevent strangulation, bowel gangrene, and poor general health condition [6].

There are cases described in the literature of patients with abdominal or inguinal hernias that discover OHs during laparoscopic repair [7]. However, there are instances in the literature that suggest the benefit of performing a prophylactic bilateral OH repair on patients. We present the case of a 79-year-old female who presented with obstructed OH that required operative treatment six months after an operation on a contralateral OH. This highlights the possible benefit of a preventative surgical approach to the contralateral side in a patient who presents with a unilateral OH.

## Case presentation

**Figure 1:**
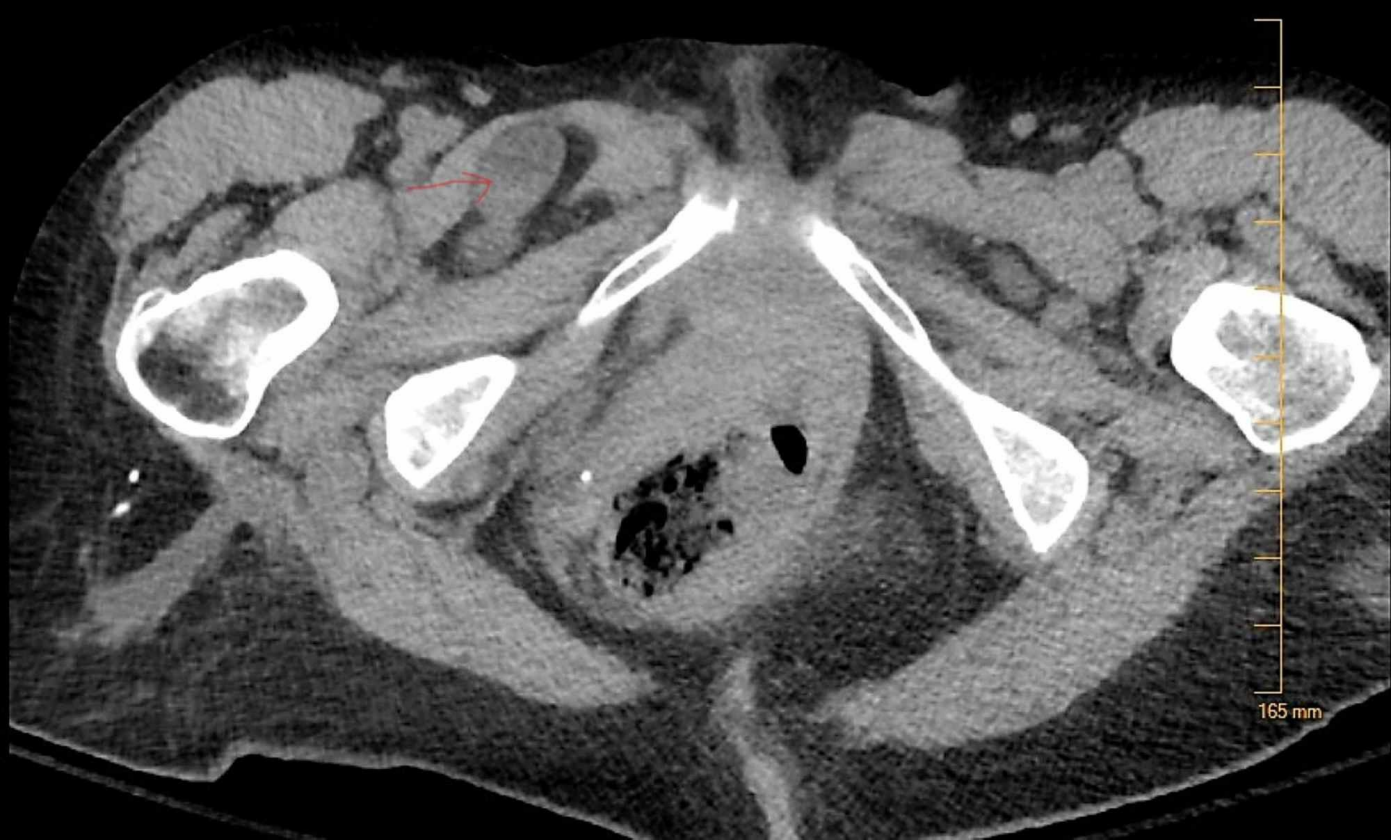
CT scan of the abdomen and pelvis showing the Right Obturator Hernia (Axial view).

**Figure 2:**
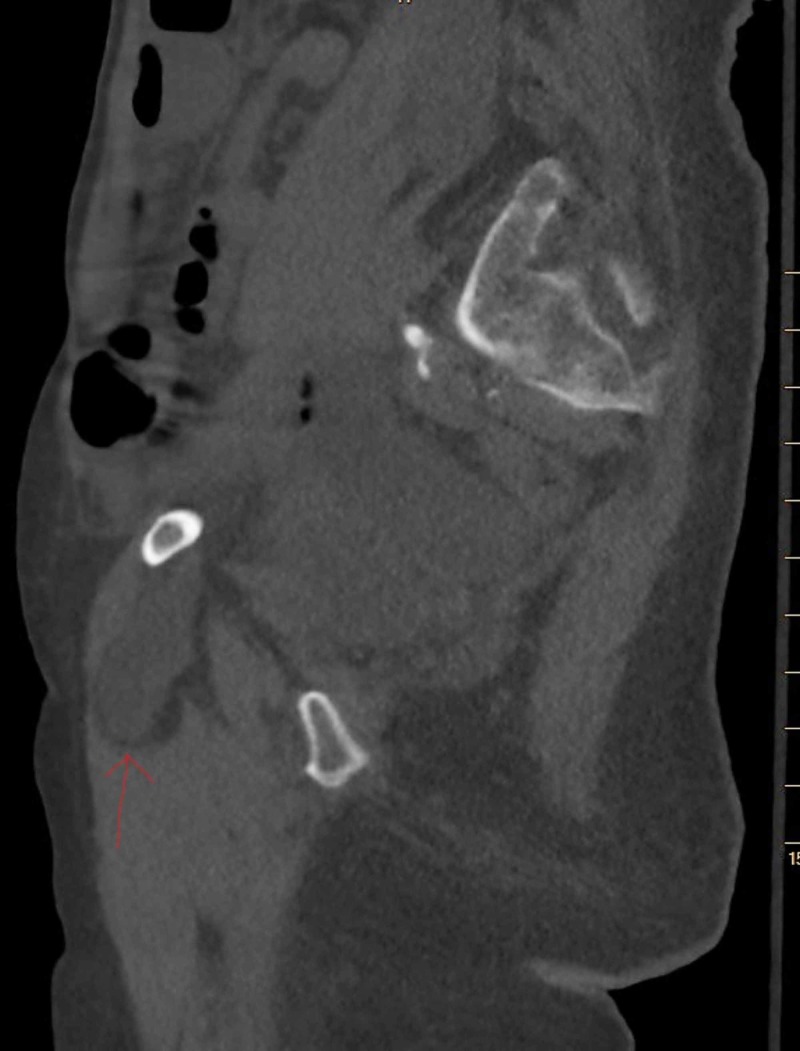
CT scan of the abdomen and pelvis showing the Right Obturator Hernia (Sagittal view).

The patient was a 79-year-old female who presented to the emergency department with an eight-hour history of abdominal pain associated with nausea and vomiting. She also complained of obstipation for the past day as well as right hip pain. Her past medical and surgical history includes right upper lobectomy for adenocarcinoma of the lung in 2008, left nephrectomy for renal cell carcinoma, prior pulmonary embolism, hypertrophic cardiomyopathy and Stage IV chronic kidney disease. Her vital signs on admission showed tachycardia and hypotension. On examination, decreased bowel sounds and mild distended was observed. A computer tomography (CT) scan of the abdomen and pelvis was performed and showed small bowel obstruction with air-fluid levels as well as a right-sided obturator hernia with a loop of small bowel which appeared incarcerated inside (Figures 1, 2). Initial laboratory studies showed mild leukocytosis with a WBC of 10.7, hemoglobin of 15 and hematocrit of 44.3. Creatinine was at a baseline level of 1.8. The patient had a nasogastric (NG) tube placed in the ER with an output of 500 cc of bilious output and was resuscitated with crystalloid and brought to the operating room.

The patient was deemed unstable to undergo a laparoscopic approach and she underwent an infraumbilical midline laparotomy through which the right-sided obturator hernia was gently reduced. The bowel was inspected and was deemed to be healthy and viable. The obturator hernia was then repaired using a polypropylene mesh plug. She had an uneventful recovery. The nasogastric tube was removed on POD 2 and the patient was started on clear liquids diet. She regained full bowel function on post-operative day 3. The patient subsequently had her diet advanced to a regular diet which she tolerated until discharge.

**Figure 3:**
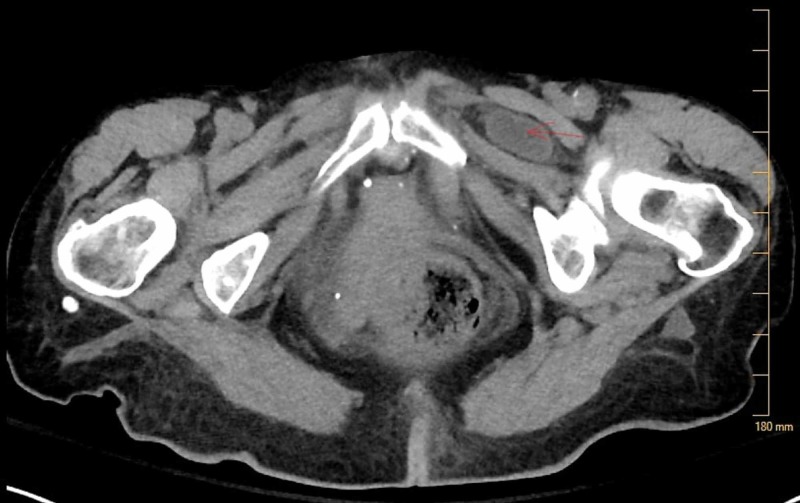
CT scan of the abdomen and pelvis showing the Left Obturator Hernia (Axial view).

**Figure 4:**
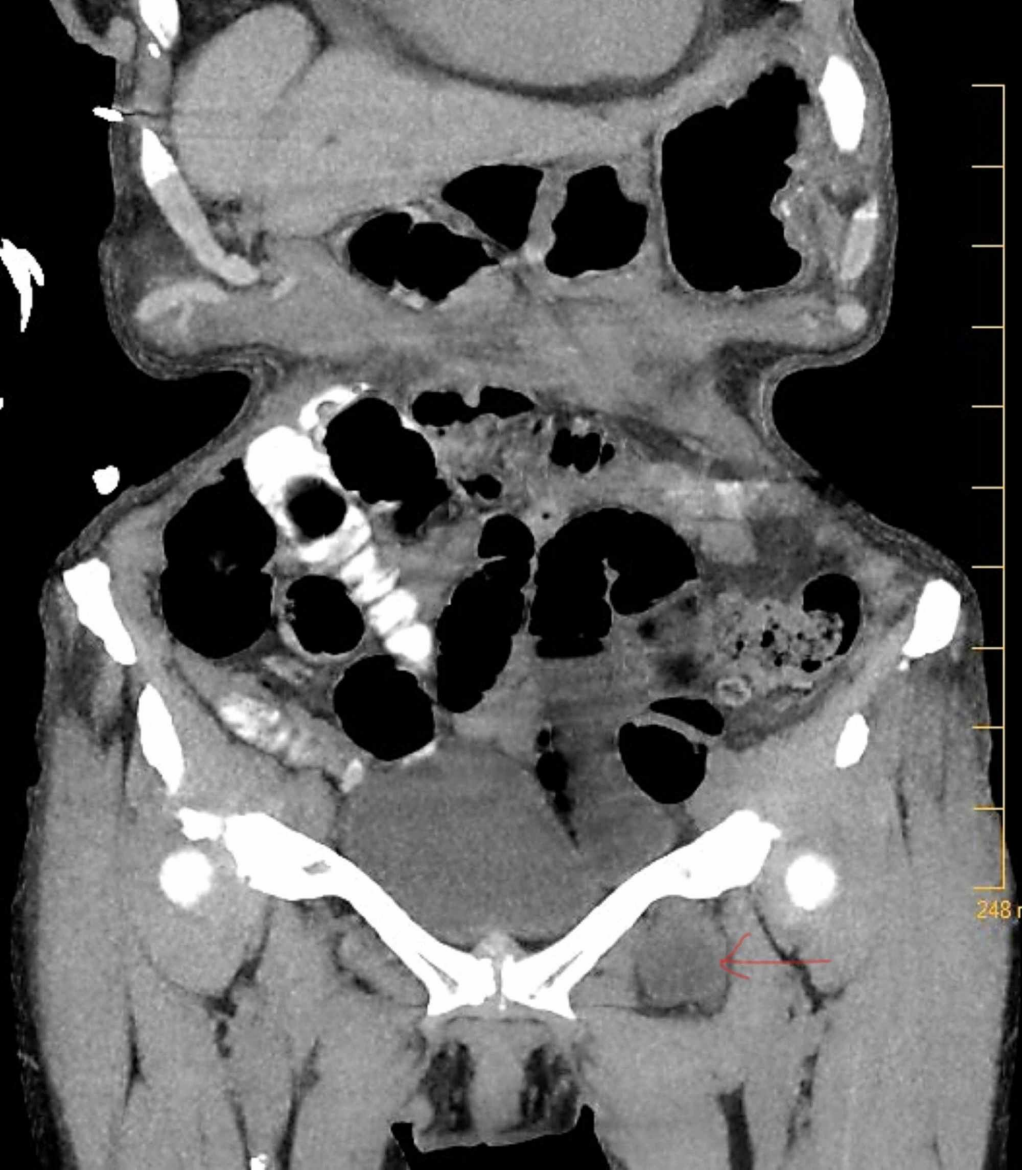
CT scan of the abdomen and pelvis showing the Left Obturator Hernia (Coronal view).

The patient returned to the emergency room six months later with the sudden onset of left hip pain associated with lower abdominal pain while walking. Soon thereafter, the patient began belching but did not develop nausea or vomiting. She underwent a CT scan of the abdomen and pelvis in the ED that demonstrated a left-sided obturator hernia as well as small bowel obstruction (Figures 3, 4). The patient was otherwise hemodynamically stable, and her laboratory studies were within normal limits. The patient was taken for an urgent midline laparotomy through her prior incision and the left obturator hernia was reduced. Intraoperatively, the bowel appeared viable and did not require any resection. The obturator hernia was repaired in a similar manner as the initial surgery with a polypropylene mesh plug. She was extubated the morning after her surgery. Recovery proceeded in a timely fashion as in her previous hospitalization. Her NG tube was removed on post-operative day 2 and she was started on a clear liquid diet. Her diet was advanced, and she remained asymptomatic until discharge.

## Discussion

OHs have the highest mortality rate of all hernias (13-40%) and are generally difficult to diagnose preoperatively [2, 8]. With insufficient clinical evidence of intestinal obstruction, a surgical approach must be considered to pinpoint the precise cause [8]. The predisposing factors for OH patients include but are not limited to: female gender, chronic disease, age, malnourishment, history of multiple pregnancies, anatomical enlargement of obturator foramen, increased intraabdominal pressure, and defective collagen metabolism [1].

One study reported the laterality of OHs (right/left/bilateral) in two groups of 41 women and two men as 11/6/6 and 9/5/6, respectively [9]. This demonstrates that the likelihood of acquiring a left-sided OH is roughly similar to that of acquiring a bilateral OH. Secondly, Susmallian et al. reported that out of 293 patients who underwent repair of bilateral or recurrent inguinal hernias, 6.82% of patients had OHs (30% unilateral, 70% bilateral) [4]. Although OHs are a rarity, the incidence of bilateral OHs is also significantly notable in patients with other inguinal hernias.

Yokoyama et al. reported out of the 659 transabdominal preperitoneal laparoscopic herniorrhaphies performed, eight of those patients were diagnosed with OH. Five of the eight patients (63%) had bilateral OHs, and seven out of the eight patients (88%) had combined groin hernias and femoral hernia [7]. Another study suggests that patients who undergo laparoscopic repair of inguinal hernias should be explored for obturator hernias as well. Approaching OHs, a lower midline laparotomy creates easy access to perform the bowel repair and view the bilateral inguinal, femoral, and obturator spaces [3]. This will also allow for other complications, such as post-operative ileus, pulmonary issues, post-surgical pain, and duration of hospital stays. Generally, early surgery or prevention will lower complication and mortality rates [3]. Susmallian et al. suggest that the incidence may be even higher if surgeons scan the pelvis in patients during laparoscopic repair [4].

Considering the difficulty of pre-operative diagnosis and high mortality rate, it may be beneficial to take preventative measures for bilateral OHs. Therefore, it could be advantageous to treat both sides when a patient presents with a unilateral OH in order to prevent delayed discovery contralaterally.

## Conclusions

Prior research has demonstrated an elevated percentage of OHs involving bilateral as opposed to unilateral presentation. It has also been suggested that the incidence of bilateral OHs would be increased further if surveillance of the pelvis intraoperatively was done routinely in hernia repairs. We present the case of a 79-year-old female who presented with an OH six months after an operation for an OH on the contralateral side. Because OHs are the abdominal hernia with the highest mortality and are difficult to diagnose preoperatively, we suggest examination of the contralateral side intraoperatively for patients presenting with unilateral OHs in order to reduce morbidity associated with a contralateral OH.
